# Altered Immunoglobulin Repertoire and Decreased IgA Somatic Hypermutation in the Gut during Chronic HIV-1 Infection

**DOI:** 10.1128/jvi.00976-22

**Published:** 2022-08-08

**Authors:** Sean T. Jones, Kejun Guo, Emily H. Cooper, Stephanie M. Dillon, Cheyret Wood, David H. Nguyen, Guannan Shen, Bradley S. Barrett, Daniel N. Frank, Miranda Kroehl, Edward N. Janoff, Katerina Kechris, Cara C. Wilson, Mario L. Santiago

**Affiliations:** a Division of Infectious Diseases, Department of Medicine, University of Colorado Anschutz Medical Campus, Aurora, Colorado, USA; b Department of Immunology and Microbiology, University of Colorado Anschutz Medical Campus, Aurora, Colorado, USA; c RNA Bioscience Initiative, University of Colorado Anschutz Medical Campus, Aurora, Colorado, USA; d Center for Innovative Design and Analysis, Department of Biostatistics and Informatics, University of Colorado Anschutz Medical Campus, Aurora, Colorado, USA; e Rocky Mountain Regional Veterans Affairs Medical Center, Aurora, Colorado, USA; Ulm University Medical Center

**Keywords:** HIV-1, IgA, gut microbiome, mucosal immunity, somatic hypermutation

## Abstract

Humoral immune perturbations contribute to pathogenic outcomes in persons with HIV-1 infection (PWH). Gut barrier dysfunction in PWH is associated with microbial translocation and alterations in microbial communities (dysbiosis), and IgA, the most abundant immunoglobulin (Ig) isotype in the gut, is involved in gut homeostasis by interacting with the microbiome. We determined the impact of HIV-1 infection on the antibody repertoire in the gastrointestinal tract by comparing Ig gene utilization and somatic hypermutation (SHM) in colon biopsies from PWH (*n* = 19) versus age and sex-matched controls (*n* = 13). We correlated these Ig parameters with clinical, immunological, microbiome and virological data. Gene signatures of enhanced B cell activation were accompanied by skewed frequencies of multiple Ig Variable genes in PWH. PWH showed decreased frequencies of SHM in IgA and possibly IgG, with a substantial loss of highly mutated IgA sequences. The decline in IgA SHM in PWH correlated with gut CD4^+^ T cell loss and inversely correlated with mucosal inflammation and microbial translocation. Diminished gut IgA SHM in PWH was driven by transversion mutations at A or T deoxynucleotides, suggesting a defect not at the AID/APOBEC3 deamination step but at later stages of IgA SHM. These results expand our understanding of humoral immune perturbations in PWH that could have important implications in understanding mucosal immune defects in individuals with chronic HIV-1 infection.

**IMPORTANCE** The gut is a major site of early HIV-1 replication and pathogenesis. Extensive CD4^+^ T cell depletion in this compartment results in a compromised epithelial barrier that facilitates the translocation of microbes into the underlying lamina propria and systemic circulation, resulting in chronic immune activation. To date, the consequences of microbial translocation on the mucosal humoral immune response (or vice versa) remains poorly integrated into the panoply of mucosal immune defects in PWH. We utilized next-generation sequencing approaches to profile the Ab repertoire and ascertain frequencies of somatic hypermutation in colon biopsies from antiretroviral therapy-naive PWH versus controls. Our findings identify perturbations in the Ab repertoire of PWH that could contribute to development or maintenance of dysbiosis. Moreover, IgA mutations significantly decreased in PWH and this was associated with adverse clinical outcomes. These data may provide insight into the mechanisms underlying impaired Ab-dependent gut homeostasis during chronic HIV-1 infection.

## INTRODUCTION

Humoral immune perturbations likely contribute to the increased susceptibility to infections and reduced efficacy of vaccines in persons with HIV-1 (PWH) (1). For logistical reasons, the majority of prior studies of humoral immune perturbations evaluated blood and lymph node specimens. However, the gastrointestinal (GI) tract is a potentially critical site for HIV-1-associated humoral immune dysfunction. HIV-1 infection is associated with a multitude of GI complications including virus-induced enteropathy and increased susceptibility to enteric pathogens such as Cryptosporidium parvum, Campylobacter, Shigella, and Salmonella (2). Extensive loss of CD4^+^ T cells occurs in the GI tract shortly after HIV-1 infection (3). Gut biopsies from acute HIV-1 infection revealed damaged follicular architecture and germinal center (GC) loss (4). In addition, the massive loss of the Th17 and Th22 subsets results in a compromised epithelial barrier, leading to the translocation of commensal bacteria and/or bacterial components such as lipopolysaccharide (LPS) from the lumen to the underlying lamina propria and the systemic circulation (5–7). This phenomenon, known as microbial translocation, is considered a major contributor to chronic immune activation, which in turn was associated with increased cardiovascular, renal, bone and other co-morbidities in PWH (8).

Homeostasis at the mucosal barrier is intricately linked to the presence of immunoglobulin A (IgA) (9), the most abundant antibody (Ab) isotype in humans. With ~2 g produced per day, IgA accounts for >80% of Ab in the mucosal compartment (10). It is composed of two subclasses, IgA1 and IgA2; IgA2 encodes a modified hinge region that makes it more resistant to bacterial endopeptidases. Compared to IgG, IgA is less inflammatory and functions through immune exclusion of pathogens, anchoring microbes into the mucus layer and masking deleterious epitopes of pathogen-derived toxins (9, 11). Studies in mice suggest that mucosal IgA coats diverse commensal bacteria to different extents (12, 13) to prevent invasion, or alternatively, to transport coated bacteria to inductive sites as a positive feedback loop for IgA production (14). Of note, several studies invoke mucosal IgA with the establishment and regulation of the microbiome (15–18). The loss of IgA class-switching in mice was linked to hyperplasia of gut lymphoid structures and altered microbial composition or dysbiosis (19). Human IgA deficiency has been associated with dysbiosis and a spectrum of GI disorders including inflammatory bowel disease (IBD) (20, 21). Thus, IgA likely plays a critical role in regulating gut microbiome homeostasis.

High-throughput sequencing has revealed that a higher fraction of Ab genes has undergone extensive somatic hypermutation (SHM) in mucosal tissues compared with peripheral blood or lymphoid tissue (22). Highly mutated IgA genes likely developed through a CD4^+^ T cell and GC-dependent process and may be critical in counteracting foreign mucosal pathogens and pathogen-derived toxins (23). In contrast, some low-affinity IgA may develop through a T-cell and GC-independent process (24) and harbor polyreactive properties that allow these IgA antibodies to bind and regulate commensal enteric bacteria (25). When present, SHM in low-affinity IgA may be mostly random and does not increase the affinity for commensal bacteria (26). Of note, high-affinity IgA monoclonal antibodies (MAbs) were isolated in normal C57BL/6 mice that regulated commensal bacterial species with putative inflammatory properties (pathobionts) (27). Moreover, microbial species coated with IgA from patients with IBD induced more inflammation in gnotobiotic mice than bacteria that were not coated with IgA (28). Thus, it is plausible that specific IgA clonotypes may be associated with specific bacterial groups or species in the microbiome.

The impact of HIV-1 infection on the complementary interactions between the microbiome, IgA antibodies and mucosal inflammation remain poorly characterized. Early studies revealed a significant decrease in IgA-producing plasma cells in the duodenum and colon of PWH compared to seronegative controls (29), but comparisons of Ab repertoire diversity were hampered by limitations in Sanger sequencing capacity. More recently, a substantial fraction of MAbs derived from the terminal ileum of PWH were shown to recognize commensal bacterial species and to be cross-reactive with Env gp41 (30), potentially skewing the early Ab response against neutralization of HIV-1 (31). Importantly, PWH were found to have defects in intestinal IgA and IgG class-switching and reduced plasma IgG responses to Proteobacteria and Firmicutes but not Bacteroidetes (32). Altogether, these findings raise further questions on the nature of the mucosal Ab repertoire in PWH in relation to the microbiome. We therefore sought to characterize the mucosal immunoglobulin (Ig) variable heavy (V_H_) and light chain (V_L_) repertoires and Ig SHM in a well-characterized, matched cohort of PWH and HIV-1-uninfected controls (33–36), and considered how these parameters relate to inflammation, the microbiome and clinical outcomes.

## RESULTS

### Chronic HIV-1 infection is associated with enhanced gut B cell activation.

**FIG 1:**
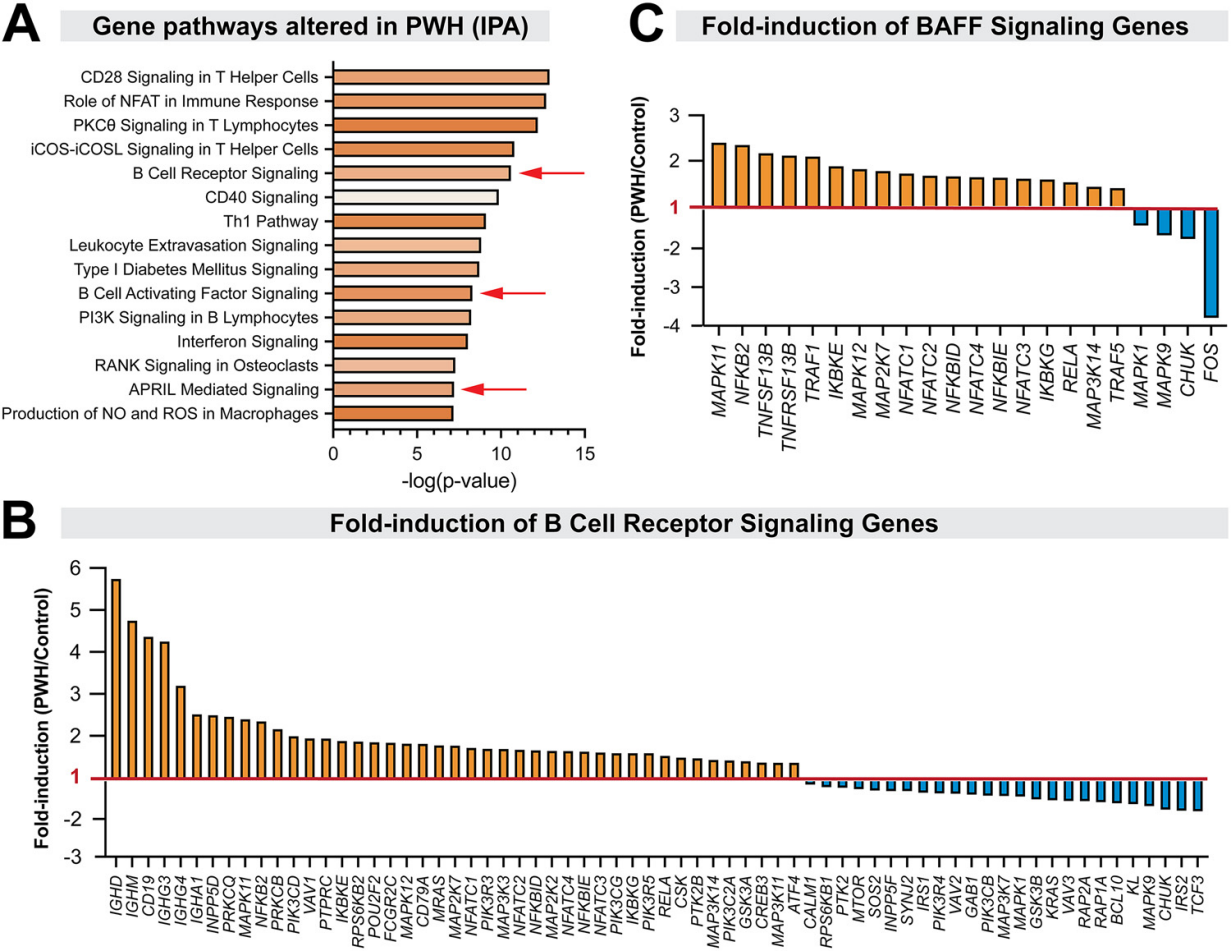
Evidence for enhanced gut B cell activation during chronic HIV-1 infection. RNAseq data were obtained from PWH (*n* = 19) and sex- and age-matched HIV-1 uninfected controls (*n* = 13). Data were normalized using the TMM method and used for ingenuity pathway analysis (IPA). (A) Gene pathways altered in PWH were ranked based on *P*-values, and the top 15 pathways were shown. Different shades of orange correspond to the magnitude of the Z-score (range: 0.16, CD40 signaling to 3.05, Role of NFAT in Immune Response). Red arrows correspond to B cell-specific pathways. Fold-induction of (B) BCR signaling and (C) BAFF signaling genes in PWH. The ratio of the average gene expression values (TMM) in PWH over controls were plotted. Orange bars correspond to genes upregulated in PWH relative to controls, whereas those in blue were downregulated. A ratio of 1 (red line) corresponds to no induction.

We previously analyzed the relationship between gut CD4^+^ T cell immunity and dysbiosis during untreated, chronic HIV-1 infection (33). B cells were not the original focus of that clinical study, and viable lymphocytes from colon pinch biopsies were no longer available for in-depth, flow cytometry-based B cell immunophenotyping. Nevertheless, RNAlater-preserved colon pinch biopsies were available from chronically-infected PWH who were not on ART (*n* = 19) and HIV-1 uninfected controls (*n* = 13) (Table 1), that allowed us to perform transcriptomic analyses (37). The cohorts were matched for age (PWH: mean 35.8 years; controls: 35.1 years, *P* > 0.05) and sex (PWH: 32% female; controls: 38% female). To determine pathways that may be altered during chronic HIV-1 infection, we subjected the Trimmed Mean of M-Values (TMM)-normalized RNAseq data for Ingenuity Pathway Analyses (41). Three B cell-specific pathways, B cell receptor (BCR), B cell activating factor (BAFF), and APRIL signaling, were significantly induced in PWH (Fig. 1A). As expected from their mechanism of action (44), curated genes under BAFF and APRIL signaling pathways were nearly identical between groups. Sixty-three percent of genes curated under the BCR signaling pathway were upregulated in PWH, with a 4.4-fold induction of *CD19* and a 2.5 to 5.7-fold induction of immunoglobulin genes (Fig. 1B; see next section). Most genes (82%) catalogued under BAFF and APRIL signaling pathways were induced in PWH, including BAFF (*TNFSF13B*) and its receptor, TACI (*TNFRSF13B*) (Fig. 1C). In addition, multiple NF-κB, NFAT, and MAP kinase genes were upregulated (Fig. 1B and C). These data suggested that chronic HIV-1 infection is associated with enhanced gut B cell activation.

**TABLE 1:** Demographics, clinical and laboratory parameters among 19 PWH and 13 control study participants

							Illumina miseq counts^*d*^							
				Blood	Colon	Plasma	IgA (V_H_)		Igκ		Igλ		IgG (V_H_)	
Code	Sex	Age	HIV^*a*^	CD4 count^*b*^	CD4 (CD45+)	Viral load^*c*^	≥2	Unique	≥2	Unique	≥2	Unique	≥2	Unique
HIV-1 uninfected individuals (controls)														
C138	M	29		728	35.48		6,190	650	87,212	5,510	310	53	2,643	269
C178	M	33		736	35.74		6,958	1,476	131,799	4,261	1,527	280	173	37
C255	M	34		588	34.27		2,990	952	119,954	3,261	225	77	154	51
C278	M	23		532	27.52		546	193	78,691	3,823	0	0	16	7
C361	F	33		720	34.68		426	126	26,427	796	5	2	9	4
C404	F	29		1,071	27.14		298	102	14,778	627	0	0	7	3
C493	F	28		672	37.26		169	59	16,949	365	187	65	2	1
C582	M	54		976	49.89		91	32	9,991	191	123	37	0	0
C708	M	47		468	32.03		1,949	615	25,716	747	30	9	61	21
C716	M	27		1,035	26.52		4,394	1306	42,575	1,459	12	6	167	38
C914	F	43		690	20.44		2,927	774	46,392	1,902	1,588	379	37	15
C947	M	25		480	25.18		2,871	691	36065	1,442	12	6	404	102
C972	F	51		1,035	22.12		2,056	562	16,246	447	12	5	0	0
Persons with HIV-1 infection (PWH)														
H124	M	48	2	400	17.21	8,400	10,615	2,672	4,701	1,139	0	0	678	206
H132	M	25	5	532	23.35	26,000	7,082	1,713	451	137	0	0	823	169
H154	F	58	6	400	10.65	22,000	11,324	2,198	1,952	466	283	56	1,140	202
H188	M	44	0.17	836	13.60	133,000	9,975	2,358	891	258	149	44	757	214
H217	M	22	1.58	744	11.35	25,200	9,534	2,347	3,061	705	198	63	120	34
H286	F	52	2.5	693	12.34	3,850	6,979	1,557	3,170	674	2,275	326	157	41
H307	M	34	0.5	624	9.36	9,180	2,544	834	12	4	12	0	345	107
H323	M	54	10	429	15.14	9440	5,183	1,306	166,415	17,334	93,268	11,686	1397	307
H391	F	29	15	238	5.63	196,000	4,156	919	103,362	14,926	177,718	27601	409	120
H428	M	28	3.25	460	13.66	25,100	3,963	908	22,727	3,992	36,828	4896	2171	423
H594	M	46	14	338	7.46	88,600	1,243	366	10,3077	12,451	99,080	12854	1018	179
H622	M	33	0.25	340	12.36	112,000	1,803	518	23,692	4,339	26,890	3042	289	60
H648	M	31	11	420	13.39	2,880	1,313	418	7,136	1,825	7,396	1214	13	6
H683	M	25	7.5	504	12.43	59,500	1,815	376	9,636	2,130	10,581	1728	68	22
H819	M	27	3.5	527	14.50	4,670	680	169	3,670	930	1,114	261	61	19
H825	M	34	1.5	364	8.81	43,200	5,101	1,334	1,366	439	1,283	324	831	145
H839	F	39	11	250	8.34	64,900	6,822	1,383	2,098	446	3,544	708	166	50
H965	F	26	0.5	221	5.21	155,000	1,658	444	5,072	1,151	2,093	425	97	24
H998	F	25	4.5	782	12.77	119,000	3,046	649	107,489	16,223	40,072	5936	607	128

a Years since first HIV-1 seropositive test.

b Blood CD4 counts correspond to the number of CD4+ T cells per μL of blood.

c Plasma viral loads correspond to copies/mL.

d Sequence reads were found at least twice within the same individual; these ‘≥2’ reads were collapsed into unique sequences to minimize the contribution of clonal expansion. Donors with <10 unique sequences were excluded for SHM analyses.

### Increased expression of gut IgA1 and other isotypes during chronic HIV-1 infection.

**FIG 2:**
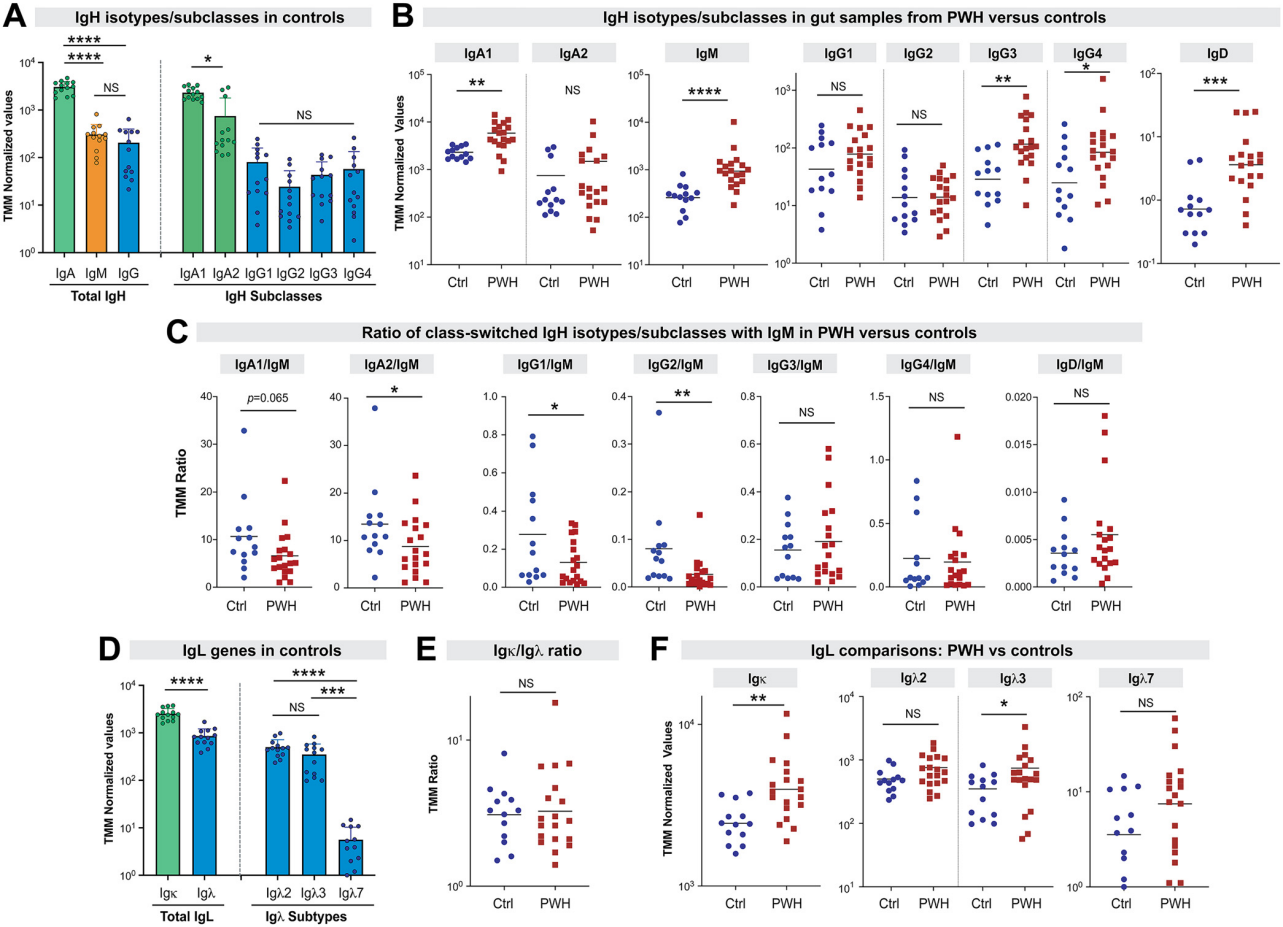
Gut immunoglobulin (Ig) isotypes, subclasses and subtypes during chronic HIV-1 infection. TMM-normalized RNAseq data were mined for Ig heavy (IgH) and light (IgL) genes. (A) Expression of IgH genes in HIV-1 uninfected controls (*n* = 13). IgA and IgG values corresponded to the sum of TMM values for individual subclasses. (B) Comparison of IgH isotypes and subclasses in PWH (*n* = 19) versus uninfected controls (Ctrl; *n* = 13). (C) Ratio of class-switched IgH isotypes/subclasses with IgM. (D) IgL genes in controls. (E) Igκ/Igλ and (F) Igκ and Igλ subtypes were compared between PWH and uninfected controls (Ctrl). All graphs were plotted on log-scale and geometric means were highlighted. Each dot corresponds to a study participant; error bars correspond to standard deviations for panels A and C. For panels A and C, multi-group comparisons were evaluated using a one-way ANOVA with pairwise comparisons using the Tukey’s honest significance difference test. For panels B to E, differences between the two groups were evaluated using a 2-tailed parametric Student's *t* test or nonparametric Mann-Whitney U test, depending on whether the sample distribution was normal. ****, *P* < 0.0001; ***, *P* < 0.001; **, *P* < 0.01; *, *P* < 0.05; NS, not significant (*P* > 0.05).

HIV-1 infection is associated with polyclonal B cell activation and hypergammaglobulinemia (4, 32, 45). Moreover, HIV-1 infection was associated with defects in class-switching based on total and antigen-specific IgA/IgM and IgG/IgM ratios in intestinal secretions (32). Given the marked induction of Ab genes under the BCR signaling pathway in PWH (Fig. 1B), we thus investigated the full set of individual Ig isotypes/subclasses in PWH versus controls. Gene expression normalized using TMM were compared. As expected, IgA comprised a large fraction of the sequence counts, 10-fold higher than IgM and 15-fold higher total IgG among controls (Fig. 2A). IgA1 was expressed at 3-fold higher levels than IgA2. Of note, the expression of IgA2 appears to be bimodal, with a small fraction of individuals expressing IgA2 to levels similar to IgA1 (Fig. 2A). Unlike in blood (~70% IgG1), no substantial differences in expression were observed between the 4 IgG subclasses. We next compared the expression of individual Ig subclasses in controls versus PWH. As shown in Fig. 2B, IgA1, IgM, IgG3 and IgG4 were significantly increased in PWH compared to uninfected controls, from 2.5-fold (IgA1) to 4.7-fold (IgM). However, no significant alterations in IgA2, IgG1 and IgG2 were observed. Although IgD was expressed at very low levels, IgD was upregulated 5.7-fold in PWH (Fig. 2B). IgE was not detected in the samples. Notably, the ratios of IgA1, IgA2, IgG1, IgG2 against IgM, but not IgG3, IgG4 and IgD, were reduced in PWH relative to controls (Fig. 2C).

Constant regions for Ig light chains (IgL) are encoded in separate chromosomes for the κ and λ light chains (chromosomes 2 and 22, respectively). Among HIV-1 uninfected controls, Igκ was more abundant, with 3-fold higher expression than Igλ, of which 3 subtypes (Igλ2, Igλ3 and Igλ7) were captured by RNAseq (Fig. 2D). It was previously reported that in the SIV-infected rhesus macaques, the ratio of Igκ and Igλ light chains may be skewed relative to uninfected controls (46). However, we did not observe significant skewing of Igκ/Igλ in PWH (Fig. 2E). Notably, Igκ was significantly increased in chronic HIV-1 infection, but of the Igλ subtypes, only Igλ3 was significantly increased (Fig. 2F). In aggregate, our data show enhanced transcription of IgA1 and select Ab subclasses/subtypes in the colon during chronic HIV-1 infection.

### Altered gut Variable (V)-gene repertoires during chronic HIV-1 infection may be linked to the microbiome.

**FIG 3:**
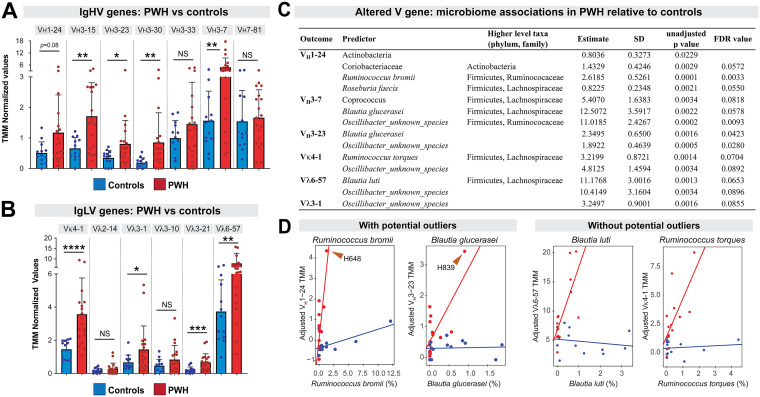
Evidence for an altered Ab repertoire in the gut during chronic HIV-1 infection. TMM-normalized RNAseq data from PWH (*n* = 19) and controls (*n* = 13) were mined for IgH and IgL V genes. (A) V_H_ gene expression PWH versus controls; (B) V_L_ gene expression in PWH versus controls. For both panels, bars correspond to mean values and error bars correspond to standard deviations. Individual data points were also shown. Differences between PWH and controls were evaluated using a 2-tailed Student's *t* test (parametric) or Mann-Whitney U test (nonparametric) depending on whether the data distribution was normal. ****, *P* < 0.0001; ***, *P* < 0.001; **, *P* < 0.01; *, *P* < 0.05; NS, not significant, though comparisons with *P*-values between 0.05 and 0.1 were noted as ‘trending’. (C) Relationships between select bacterial taxa relative abundance (RA) and V gene expression that are significantly different between PWH and controls. Microbiota RA (%) was regressed on V gene expression (adjusted TMM) with an HIV-1, V gene interaction term and adjusting for age, sex and HIV status. All associations are significant based on FDR < 10% except for Actinobacteria (*P *> 0.1) where no adjustments were made for multiple comparisons due to the small number (*n* = 4) of phyla tested. (D) Select significant relationships for bacterial taxa in (C) are shown. Visual inspection suggested potential study participant outliers in some (left) but not all (right) associations.

The human Ig locus encodes 36–49 functional V_H_ and 64–65 functional V_L_ (35 Vκ and 29–30 Vλ) genes (42), from which only one V_H_ and one V_L_ are selected for each mature B cell/Ab molecule. The prevalence of V gene transcripts in a particular sample thus provides a measure of Ab repertoire diversity. We detected 42 V genes (21 V_H_, 6 Vκ and 15 Vλ) in the data set. TMM-based normalization removed low-count genes earlier in the analytical pipeline. This narrowed our analysis to 13 V genes (Fig. 3), which were either among the most abundantly expressed in the gut or the most easily mapped to the reference human genome. Several V genes were expressed at higher levels in PWH (Fig. 3). Altered V-genes included V_H_3-15, V_H_3-23, V_H_3-30, and V_H_3-7 (Fig. 3A), as well as Vκ4-1, Vλ3-1, Vλ3-21, and Vλ7-81 (Fig. 3B). Of note, increased V gene expression in PWH was not observed for all V genes: V_H_3-33, V_H_7-81, Vλ2-14 and Vλ3-10 were similarly expressed in PWH versus controls (Fig. 3), arguing against a global effect of increased V gene expression. These data indicate significant perturbations in the gut Ab repertoire during chronic HIV-1 infection.

We hypothesized that, if the gut Ab repertoire evolved to regulate the microbiome (25), then the expression of certain V genes may track with the relative abundance (RA) of select bacterial species, and these associations may differ between controls and PWH. Because microbiome data has been obtained previously (33–36), we regressed V gene expression on RA of prevalent microbial taxa for the entire cohort, with an interaction term between microbiome RA and HIV-1 status, adjusting for age, sex and HIV-1 status. Regression slopes (estimates) revealed 14 V gene-microbiome taxon associations in PWH but not controls (Fig. 3C). Visual inspection suggested that some associations may be driven by outlier individuals, but this did not apply to all associations (Fig. 3D). These analyses suggest that chronic HIV-1 infection may alter the nature of interactions between mucosal antibodies and the gut microbiome.

### Decreased mucosal IgA SHM in chronic HIV-1 infection.

**FIG 4:**
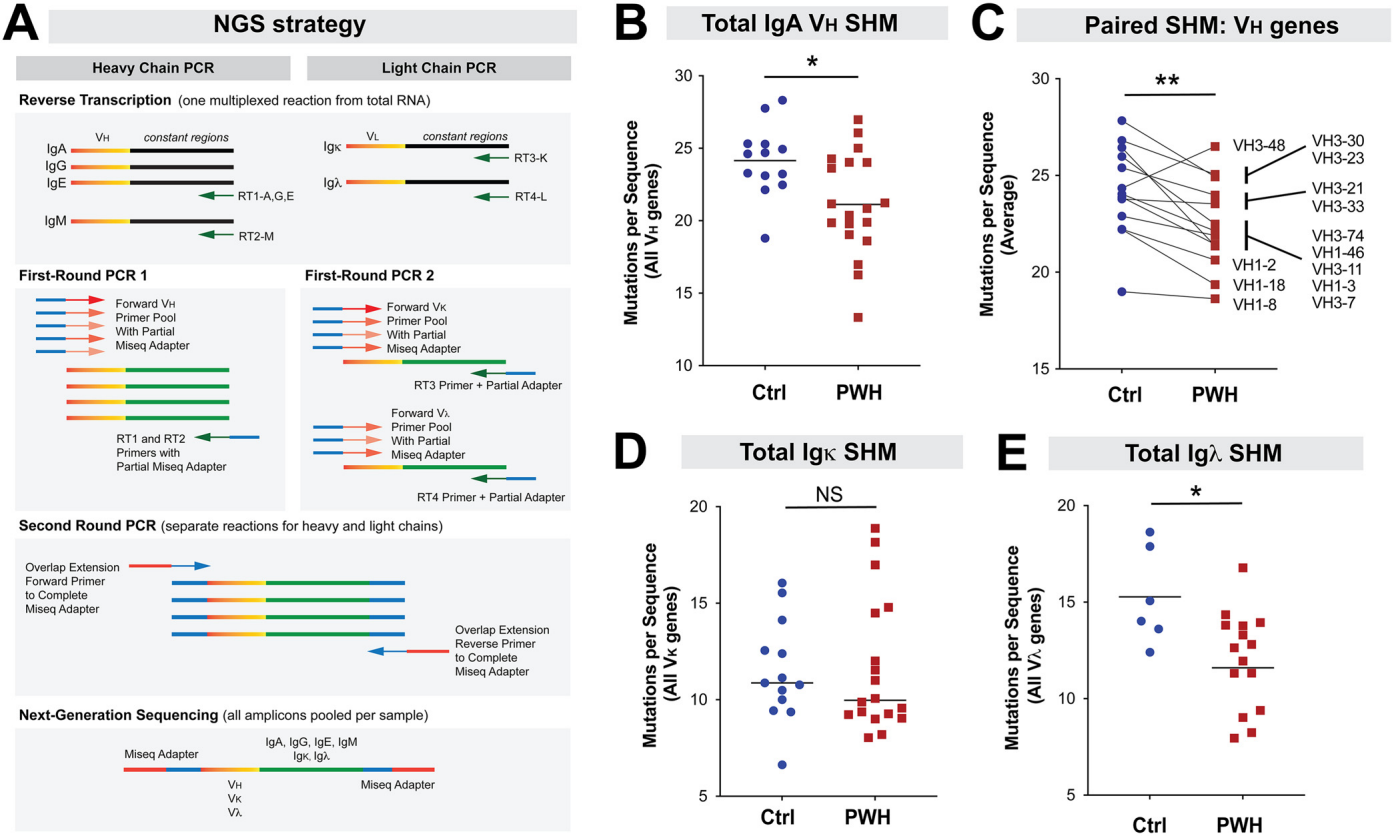
Decreased gut Ig SHM during chronic HIV-1 infection. (A) NGS approach. Reverse transcription was performed using pooled primers specific for IgA/IgG/IgE, IgM, Igκ and Igλ. The cDNA was then used for 2 separate multiplex first-round PCRs for V_H_ and V_L_. In the second round PCR, overlap extension primers were used to complete the Miseq Adapter. Per donor, only sequences found at least twice were analyzed. Identical sequences were collapsed into unique sequences as noted in Table 1, and the number of mutations per germ line V sequence based on IMGT were calculated. (B) The overall SHM of IgA sequences were computed, each dot corresponds to a donor. (C) Mean mutation rates for abundant V_H_ genes were compared between PWH and controls. Abundant V_H_ genes correspond to those that were represented with >10 unique sequences in at least 5 PWH and 5 control donors. Most V_H_ genes, except V_H_3-48, had decreased SHM in PWH relative to controls. (D) Overall SHM in Igκ sequences. Note that donor H307 (PWH) was removed due to insufficient number of sequences (<10; Table 1). (E) Overall SHM in Igλ sequences. Due to low sequence counts, 3 PWH and 7 controls were excluded from the analysis (Table 1). In Panels B, D and E, the differences between PWH and controls were evaluated using a 2-tailed unpaired Student's *t* test (parametric) or Mann-Whitney U test (nonparametric) depending on whether the data distribution were normal or skewed, respectively. In panel C, differences were evaluated using a 2-tailed paired Student's *t* test. ****, *P* < 0.0001; ***, *P* < 0.001; **, *P* < 0.01; *, *P* < 0.05; NS, not significant.

The extent of SHM in the antigen-binding region typically correlate with the strength of Ab binding and their function. Thus, profiles of somatic hypermutation (SHM) in immunoglobulin genes may delineate T-dependent versus T-independent IgA that may be important in counteracting foreign mucosal pathogens versus regulating commensal bacteria, respectively (11, 23–25). To date, the extent of SHM in gut IgA genes during chronic HIV-1 infection relative to controls remains unclear. Thus, we designed forward primers that would capture potentially more abundant V_H_ and V_L_ genes in Fig. 3 and linked them to Illumina Mi-Seq adaptors (Fig. 4A). Of note, these primer sets could also capture less abundant V genes that were similar in sequence to the prevalent ones. Reverse primers were designed to amplify IgA (Cα gene), IgK (Cκ), and Igλ (Cλ). The primer combinations would result in amplicons that span the entire VDJ (IgH) and VJ (IgL) genes. We discarded sequences found only once in the sample as it would significantly reduce the error rate of the assay (47, 48). To minimize the effects of clonal expansion, we also ‘collapsed’ identical sequences for each donor into a single ‘unique’ sequence. Table 1 shows the number of filtered (≥2/donor) and unique sequences analyzed for each donor. For IgH, we obtained a median of 732 unique sequences (range: 32–2,672) per donor, whereas Igκ had a median of 1,145 (range: 4–17,334). We obtained a much lower sequence yield for Igλ, with 71 sequences/donor, with 10 donors yielding <10 unique sequences each.

IgA SHM was calculated against germ line V genes minus the complementarity determining region 3 (CDR3), which is difficult to map to the corresponding V(D)J germ line genes. Using all unique V_H_ sequences captured per donor, we observed a significant decrease in overall IgA V_H_ SHM in PWH compared to controls (Fig. 4B). However, not all V_H_ genes were found in all donors. Paired analyses of the 13 most abundant V_H_ sequences captured also revealed an overall decrease in SHM (Fig. 4C). Overall mutation rates for Igκ were not significantly lower in PWH versus controls (Fig. 4D). By contrast, decreased Igλ SHM was observed in PWH (Fig. 4E), with the caveat that 10 donors (3 PWH, 7 controls) were not included in the analysis (Table 1). Overall, chronic HIV-1 infection is associated with a significant decrease in gut IgA SHM.

### Highly mutated IgA sequences are depleted in the gut during chronic HIV-1 infection.

**FIG 5:**
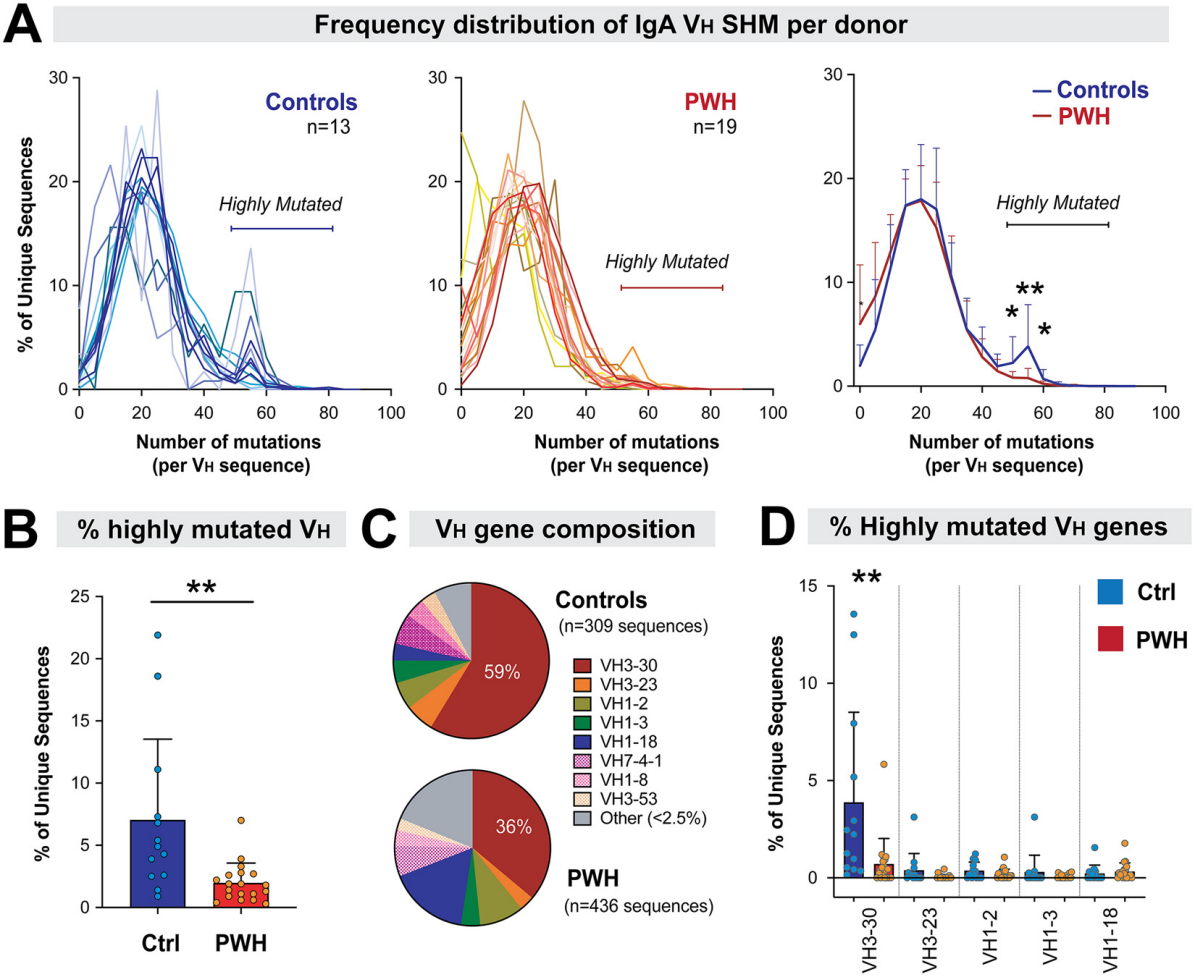
Loss of highly mutated (≥50 mutations/sequence) gut IgA during chronic HIV-1 infection. (A) Frequency of highly mutated sequences in (left) controls versus (middle) PWH. Each line corresponds to the percentage of unique sequences with a specific number of mutations per donor. The average number of sequences with 0–5, 5–10, 10–15, etc. mutations were plotted to compare controls versus PWH (right). Error bars correspond to SD. Only the statistically significant differences based on a 2-tailed Mann-Whitney U test were shown. (B) The percentage of highly mutated unique sequences were compared between PWH and controls. (C) V_H_ gene composition. Highly mutated sequences were pooled for controls (*n* = 309 sequences) and PWH (*n* = 436 sequences) and V_H_ gene percentages were calculated. (D) The percentage of highly mutated sequences per donor were computed for the most represented V_H_ genes in the data set. Donors without these V_H_ genes were excluded. For panels B and C, comparisons were made using a 2-tailed Mann-Whitney U test or Student's *t* test depending on whether the data were skewed or normal, respectively. Comparisons without an asterisk were not significant. **, *P* < 0.01; *, *P* < 0.05.

It was previously noted that some IgA sequences could have very high mutation rates (22). We were interested in determining if heavily-mutated IgA sequences are increased in HIV-1 infection, as most broadly-neutralizing antibodies (bNAbs), a critical goal for HIV-1 vaccine development, have high mutation rates (49, 50). On average, the majority of IgA V_H_ sequences in HIV-1 uninfected controls had ~20 nucleotide mutations (Fig. 5A, left panel), consistent with a previous study that sequenced 196 IgA+ ileal B cells (25). However, 7.0% of the IgA V_H_ sequences had ≥50 mutations, which corresponds to ~15% mutation rate. Interestingly, the proportion of these highly mutated sequences (≥50 mutations) drastically declined in PWH to 2.0% (Fig. 5A, middle panel), and these differences were statistically significant (Fig. 5A, right panel and Fig. 5B). V_H_3-30 was the most abundant V_H_ gene among highly mutated sequences, accounting for 59% and 36% of highly mutated sequences in controls and PWH, respectively (Fig. 5C). The key difference in SHM of IgA between groups was the paucity of highly mutated sequences among PWH; these differences were driven largely by SHM frequencies in V_H_3-30 (Fig. 5D).

### Transversion mutations account for decreased IgA SHM in chronic mucosal HIV-1 infection.

**FIG 6:**
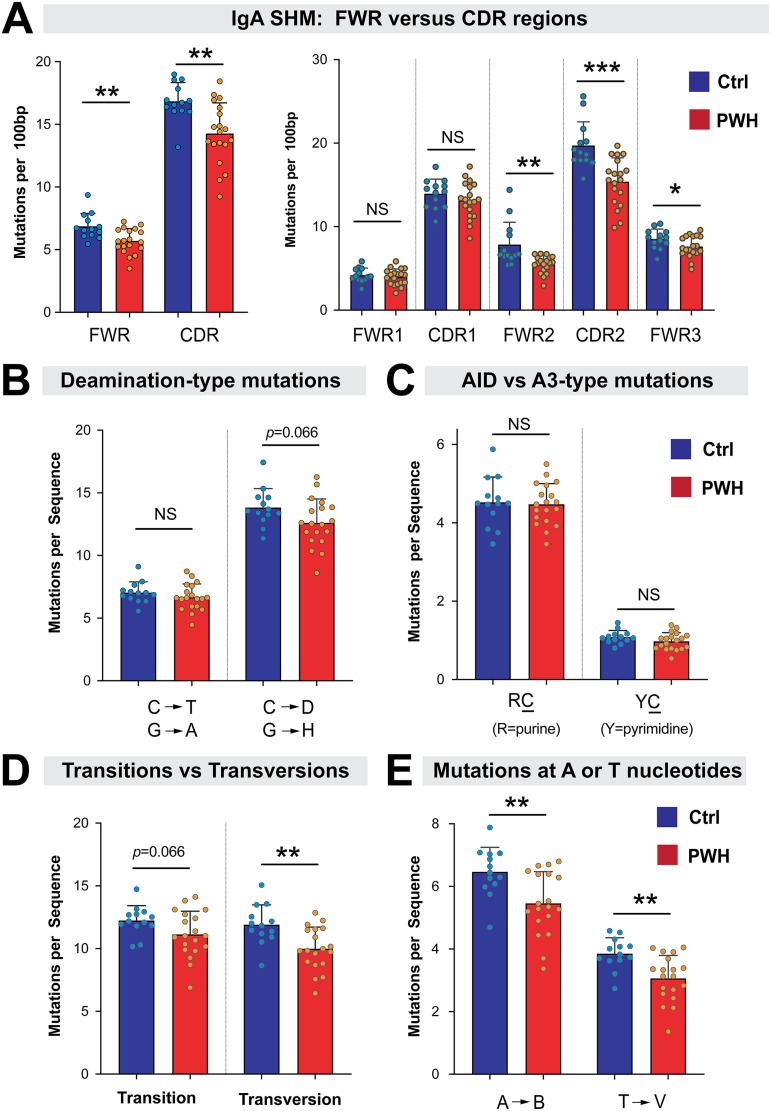
Mutational contexts of gut IgA SHM in PWH versus controls. (A) SHM in framework (FWR) versus complementarity-determining regions (CDRs). (*Left*) Mutations per 100 bp of sequence were tabulated for the entire FWR and CDR regions for PWH (red) and controls (blue). (*Right*) Mutation rates were determined for distinct FWR and CDR segments. (B) Deamination-type mutations were counted for individual donors. (C) Mutations at dinucleotide contexts that are considered as ‘hot spots’ for deamination by AID (RC; where R = purine) or APOBEC3 (YC, where Y = pyrimidine) were evaluated for each donor. Note that C corresponds to the mutated deoxycytidine, and the calculations also accounted for the opposite strand, which would be GY for AID-type and GR for APOBEC3-type mutations. (D) Comparison of transition (R→R’; Y→Y’) versus transversion (R↔Y) mutations. (E) Comparison of mutations at A or T nucleotides in PWH versus controls. B= deoxynucleotides other than A; V = deoxynucleotides other than T. In all panels, bars correspond to means and error bars are SD. Circles correspond to data from individual donors. Differences between controls and PWH were evaluated using a 2-tailed Student's *t* test or Mann-Whitney U test depending on whether the data distribution was normal or skewed, respectively. *, *P* < 0.05; **, *P* < 0.01; ***, *P* < 0.001; NS, not significant. *P*-values <0.1 were explicitly noted.

We next determined the location of IgA mutations that were decreased in HIV-1 infection. The antigen-binding site of Ab is usually formed by the structural juxtaposition of the CDR1, CDR2, and CDR3 regions. Framework (FWR) regions harbor fewer mutations as these encode domains that stabilize the Ab, but in HIV-1 bNAbs, these regions are also heavily mutated (49). Our analyses revealed that SHM in the V_H_ (minus CDR3) gene was decreased in both the CDR and FWR regions in PWH relative to controls (Fig. 6A, left panel), particularly in CDR2, FWR2 and FWR3 (Fig. 6A, right panel).

Ab mutations are initiated by activation-induced deaminase (AID), which converts cytidines to uridines, resulting in a C→T and on the opposite strand, G→A mutations (51). The uracil bases can be excised by deglycosidases and replaced by error-prone polymerases. Thus, C→D (where D is any base other than C) or G→H (where H = any base other than G) mutations could be directly attributed to AID plus error-prone polymerases. Recently, we obtained evidence that the APOBEC3 deaminases may complement AID in inducing Ig SHM (47, 48). In contrast to AID, which preferentially mutates deoxycytidines preceded by a purine (RC, where R = purine), the APOBEC3 deaminases preferentially mutate deoxycytidines preceded by a pyrimidine (YC, where Y = pyrimidine). Interestingly, IgA deamination mutations were not significantly decreased in PWH relative to uninfected controls (Fig. 6B), even after subdividing into AID-type (RC) versus APOBEC3-type (YC) mutations (Fig. 6C). Consistent with these findings, we did not observe a difference in transition (R→R’; Y→Y’) mutations in PWH (Fig. 6D). Instead, we observed a significant decrease in transversion (R↔Y) mutations (Fig. 6D). The IgA mutations that decreased in PWH mainly occurred in A and T nucleotides (Fig. 6E). Altogether, our findings reveal that the decrease in overall IgA SHM in PWH were due to a decrease in transversion mutations and mutations at A and T nucleotides. These findings suggest that chronic HIV-1 infection may significantly impact IgA SHM downstream of AID/APOBEC3 deaminase function.

### Decreased mucosal IgG SHM in chronic HIV-1 infection.

**FIG 7:**
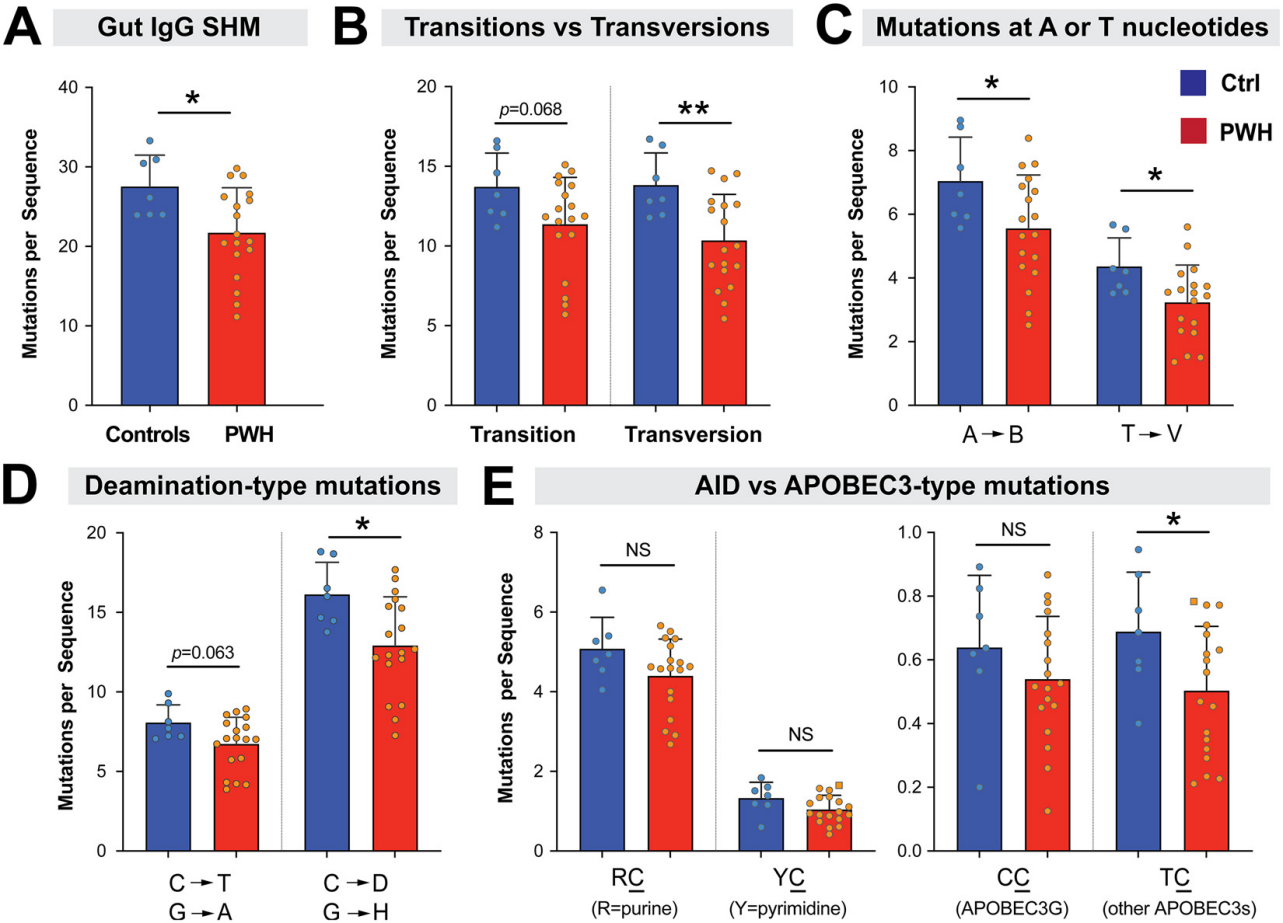
Decreased mucosal IgG SHM in PWH. Unique IgG sequences were pooled for each donor and mutational rates determined. 6 PWH and 1 control were excluded due to low numbers of sequences (<10; Table 1). (A) The overall SHM of IgG sequences were computed. (B) Comparison of transition (R→R’; Y→Y’) versus transversion (R↔Y) mutations. (C) Comparison of mutations at A or T nucleotides in PWH versus controls. B= deoxynucleotides other than A; V = deoxynucleotides other than T. (D) Deamination-type mutations were counted for individual donors. (E) Mutations at dinucleotide contexts that are considered as ‘hot spots’ for deamination by AID (RC; where R = purine) or APOBEC3 (YC, where Y = pyrimidine; subdivided into APOBEC3G-type CC or other APOBEC3s TC) were evaluated for each donor. Note that C corresponds to the mutated deoxycytidine. The calculations also accounted for the opposite strand, which would be GY for AID-type and GR for APOBEC3-type mutations. In all panels, bars correspond to means and error bars are SD. Circles correspond to data from individual donors. Differences between controls and PWH were evaluated using a 2-tailed Student's *t* test or Mann-Whitney U test depending on whether the data distribution was normal or skewed, respectively. *, *P* < 0.05; **, *P* < 0.01; ***, *P* < 0.001; NS, not significant.

Our analyses have focused on IgA as it was the most dominant isotype in the colon biopsies. However, IgG also plays important roles in mucosal immunity (52). On average, 10.6% of Ab sequences recovered per donor were IgG, which included IgG1 (7.6%), IgG2 (2.0%), IgG3 (0.86%) and IgG4 (0.078%). Thus, we also compared the relative levels of IgG SHM between PWH and controls. Sequences from the four IgG subclasses were pooled. Using similar filtering criteria for IgA SHM analyses, seven individuals (6 controls, 1 PWH) were excluded from the analysis due to low sequence count (Table 1). We observed a significant decrease in gut IgG SHM in PWH relative to controls (Fig. 7A). Similar to our results with IgA, the decrease of IgG SHM in PWH was linked to transversion mutations (Fig. 7B), particularly mutations at A and T nucleotides (Fig. 7C). However, in contrast to the IgA SHM results, we also observed a decrease in transition and deamination-type mutations, though these differences did not reach statistical significance (*P *=* *0.06; Fig. 7B and D). Specifically, there was a significant decrease in mutations at C or G nucleotides (Fig. 7D). We next tested if these C or G mutations fall into any AID-type or APOBEC3-type contexts, and found a significant difference in TC mutations, which have been linked to APOBEC3 deaminases other than APOBEC3G (47). With the caveat that IgG sequences had dramatically lower abundance than IgA in the gut, these data suggest that chronic HIV-1 infection also impaired mucosal IgG SHM.

### Decreased IgA SHM is linked to systemic and mucosal CD4^+^ T cell loss during in HIV-1 infection.

**FIG 8:**
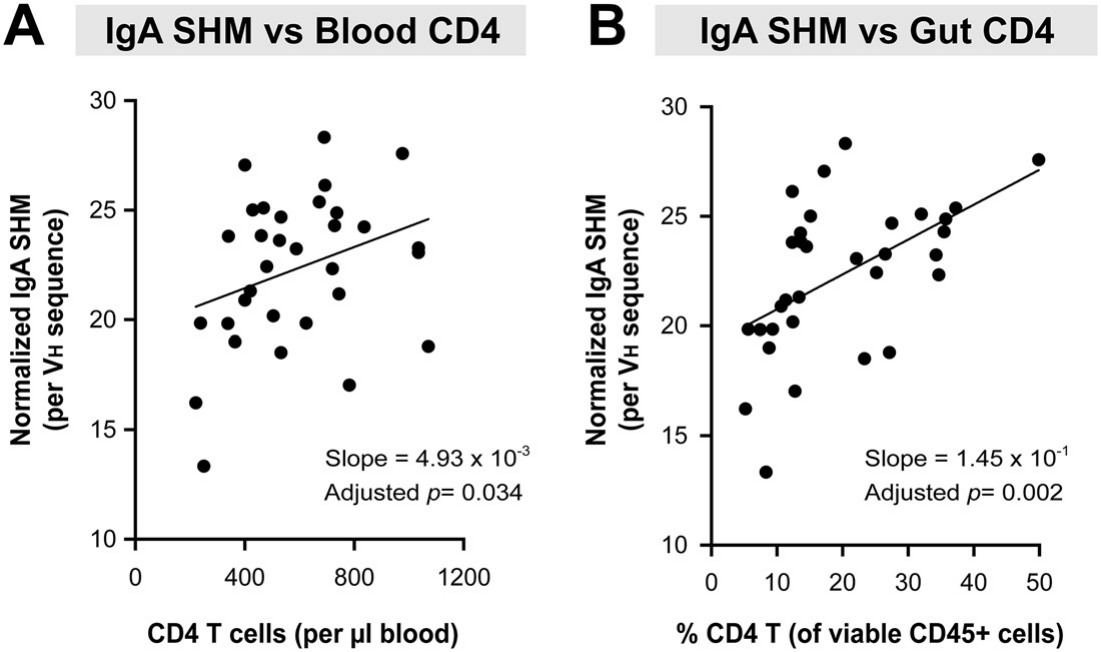
IgA SHM is correlates with CD4 T cell levels in the blood and the gut. The relationship between overall IgA V_H_ SHM rates in the gut were compared to 14 clinical, immunological and microbial parameters while controlling for age and sex. (A) CD4 T cell count in the blood and (B) the percentage of CD4^+^ T cells within viable CD45^+^ lymphocytes in the gut positively correlated with IgA SHM rates. Slopes and adjusted *P*-values are noted. Other significant correlations were listed in Table 2.

Canonical SHM is linked to GC development (15). However, SHM may also occur in a T-cell and GC-independent manner. The cohort studied exhibited significant CD4^+^ T cell loss in PWH using a 2-tailed Mann-Whitney U test, both in the blood (PWH median 429 cells/μL vs controls 720, *P *<* *0.002) and the gut (PWH median % of viable CD45^+^ T cells 12.4% vs controls 32.0%, *P *<* *0.0001). Given the loss in CD4 T cells in both compartments in PWH relative to controls, we determined whether the decrease in IgA SHM (Fig. 4B) was linked to CD4^+^ T cell levels as well as 12 other clinical, immunological, and virological parameters obtained from previous studies (33–36) after adjusting the data for sex and age. These included gut CD8^+^ T cell counts/frequencies, virological measures (gut HIV-1 RNA and plasma viral loads), and markers of inflammation (IL-6, CRP), microbial translocation (LPS, sCD14, LTA), immune activation (CD38^+^ HLA-DR+ CD4 and CD8 T cells, sCD27) and mucosal barrier dysfunction (iFABP). IgA SHM positively correlated with higher CD4^+^ T cell counts in the blood (Fig. 8A) and more significantly, in the gut (Fig. 8B). Lower IgA SHM was also associated with increased systemic immune activation and microbial translocation (Table 2). Thus, the loss of IgA SHM in the gut is associated with features of mucosal HIV-1 immunopathology.

**TABLE 2:** Association between IgA SHM versus clinical and immunological parameters^*a*^

Compartment	Parameter	Slope^*b*^	Adjusted P^*c*^
Blood	Blood CD4 count (cells/μL)	0.0049	0.0336
Blood	Plasma sCD27 (U/mL)	-0.0964	0.0019
Blood	Plasma LPS (pg/mL)	-0.2757	0.0151
Gut	Gut CD38+HLA-DR+ CD4 T cells	-0.5492	0.0222
Gut	Gut CD38+HLA-DR+ CD8 T cells	-0.1044	0.0433
Gut	Gut CD4 T cells (% of viable CD45+ cells)	0.1453	0.0018
Gut	CD8 T cells (% of viable CD45+ cells)	-0.1131	0.0010

a Multivariate linear models to predict IgA SHM based on clinical and immunological parameters, adjusting for age and sex.

b Represents the direction of the linear association between IgA SHM and the parameter, when adjusting for age and sex. A negative slope represents an inverse relationship between IgA SHM and the parameter. A positive slope represents a direct relationship between IgA SHM and the parameter.

c *P*-value were adjusted using the Benjamini and Hochberg False Discover Rate to account for multiple testing.

## DISCUSSION

HIV-1 infection is associated with a multitude of B cell abnormalities that include alterations in the Ab repertoire, perturbations of B cell subpopulations and loss of GCs in the GI tract (4, 29). How these humoral immune perturbations associate with inflammation and dysbiosis remains unclear. We compared the Ab repertoire in colon biopsy samples from a cohort of PWH who were not on ART versus age- and sex-matched HIV-1-uninfected controls. The cohort is unique as virological, immunological, microbiome and clinical data were previously compiled for these individuals, which allowed for testing potential correlations with the Ab profiles obtained. We highlight two major findings pertaining to Ab profiles in the GI tract of this cohort. Relative to controls, colon biopsies from PWH exhibited: i) a significantly altered Ab repertoire and ii) significantly decreased rates of Ig SHM. Further, these findings associated with specific microbial constituents and markers of mucosal immune dysfunction/inflammation, respectively.

### Association of alterations in the gut Ab repertoire in PWH with the microbiome.

Differences in the percentage of certain V genes in PWH relative to controls suggested an altered gut Ab repertoire during chronic HIV-1 infection. This altered Ab repertoire coincided with increased B cell activation, increased IgA1, IgM, IgG3, IgG4, and IgD expression, and decreased class-switching to IgA1, IgA2, IgG1, and IgG2 in the GI tract of PWH. Previous studies revealed a modest but significant decrease in IgA-producing plasma cells in the colon of PWH (29) and in gut tissues of SIV-infected nonhuman primates, despite increased expression of B cell survival factors (53). Thus, one explanation for the altered gut Ab repertoire was that HIV-1 infection resulted in the disproportionate expansion of select B cell clones, whereas other B cell clones were deleted. To date, there is limited information on how interactions between the mucosal Ab repertoire and the microbiome are altered during chronic HIV-1 infection. Using regression approaches, we identified associations between V genes and certain microbiome taxons that were more significant in PWH versus controls. These relationships included not only commensal bacterial taxa that may be important in host health (Lachnospiraceae and Ruminococcaceae), but also bacterial taxa that have been linked to pathophysiology (Coriobacteriaceae) (54–56). Moreover, several V genes were associated with multiple bacterial taxa, consistent with the reported polyreactivity of human IgA monoclonal antibodies derived from stool (57, 58). Notably, we were unable to identify significant Ab-microbiome associations in controls that were disrupted in PWH. This may be due to the limited sample size and stringency of the statistical approach. Less stringent approaches, such as separate regression analyses for controls, identified significant V_H_3-7:Proteobacteria and Vλ3-1:Verrucomicrobia associations that were not detected in PWH. Notably, decreased plasma IgG reactivity to Proteobacteria was observed in PWH (32). Thus, the current work may guide subsequent statistically-powered clinical studies. Importantly, the current associations do not provide cause-and-effect information. Follow-up investigations using recombinant Abs from PWH in experimental systems such as gnotobiotic mice (18) may help unravel how these mucosal Ab-microbiome associations in PWH affect pathogenic outcomes.

### Chronic HIV-1 infection is associated with a significant decrease in IgA SHM.

Prior to this work, the impact of HIV-1 infection on the frequencies of SHM in the GI tract remained unclear, as NGS approaches have yet to be implemented. Initial Ab responses against HIV-1 may originate from B cell precursors in the gut (30, 59), and the gut may provide a repository for Abs with high levels of Ig SHM (22). Notably, a major goal of HIV-1 vaccine development is to elicit broadly-neutralizing antibodies (bNAbs), most of which exhibit very high levels of SHM. AID-mediated SHM is highly dependent on CD4^+^ T cell help, but data on acute SIV infection of nonhuman primates suggest prolonged survival of follicular CD4^+^ T cells in inductive sites in the gut (53). By contrast, in mice, IgA SHM was highly dependent on RORγT (15), which controls Th17 differentiation. Th17 cells are significantly depleted early during HIV-1 infection, and likely contribute to barrier dysfunction. We observed significantly lower IgA SHM in the gut of PWH compared to controls, and this decrease in SHM significantly correlated with lower frequencies of gut CD4^+^ T cells in PWH. These data suggest that high-affinity mucosal IgA+ B cells may be depleted during HIV-1 infection. A loss of high-affinity IgA+ B cells could contribute to increased susceptibility of PWH to enteric pathogens such as Cryptosporidium, Salmonella, Campylobacter, and Shigella (2). Interestingly, Igκ SHM appeared relatively unperturbed in PWH, but not Igλ SHM. As our NGS approach relied on bulk sequencing, it is unclear what proportion of these light chain sequences were linked to IgA, IgG, or IgM. Follow-up studies using paired V_H_-V_L_ sequences using single-cell RNAseq may improve the granularity of IgL SHM data sets.

Understanding the mechanisms underlying lower IgA SHM may have implications for addressing gastrointestinal infections among PWH. The decline in IgA SHM in PWH relative to controls were primarily due to transversion mutations that occurred at A or T nucleotides. Thus, the defect in Ig SHM in the gut may not be due to decreased function of AID or even the APOBEC3 deaminases, which primarily mutate at C or G nucleotides. Mutations at A or T nucleotides in Ig loci stem from the error-prone gap repair facilitated by MutSα, which removes stretches of deoxynucleotides containing uracils, and filled in by error-prone polymerases such as Polη (60, 61). Recently, the SAMHD1 restriction factor has been associated with Ig transversion mutations in B cells (62). SAMHD1 is a dNTPase that can skew intracellular dNTP pools, and its activity *in vivo* may be regulated by lipopolysaccharide (LPS) (63, 64). Given that HIV-1 infection results in mucosal barrier dysfunction and the translocation of microbes and microbial products (including LPS), we hypothesize that SAMHD1 may contribute to these defects in IgA SHM. In fact, the decrease in IgA SHM that we observed correlated with markers of microbial translocation and T cell activation. It would be of interest to investigate perturbations in error-prone gap repair and SAMHD1 in gut B cells during chronic HIV-1 infection in follow-up work. Of note, we also observed significantly lower gut IgG SHM in PWH versus controls. In addition to decreased transversion mutations, we detected decreased mutations at C or G nucleotides in the TC context, which is associated with APOBEC3 deamination. However, due to the low numbers of gut IgG sequences recovered, these results remain tentative and would require confirmation. It would also be important to evaluate if these defects occur during antiretroviral therapy, which do not completely restore the mucosal barrier during HIV-1 infection.

### Conclusions.

The current study highlights Ab repertoire changes and decreased IgA SHM in colon biopsies from untreated PWH. Regression analyses raise the possibility that these Ab repertoire perturbations may have consequences for homeostatic control of the microbiome. Moreover, decreased IgA SHM in PWH was associated with CD4^+^ T cell loss. Further studies on the relationship between the gut Ab repertoire, Ig SHM, barrier integrity and the microbiome may provide insights for restoring mucosal immune responses in PWH.

## MATERIALS AND METHODS

### Clinical cohort.

Archived specimens were obtained from a completed clinical study (33) involving 19 persons with HIV-1 (PWH) and 13 age- and sex-matched controls that was previously approved by the Colorado Multiple Institutional Review Board. On average, PWH had been infected (defined by the first HIV-1 seropositive test) for 5.25 ± 1.1 (SEM) years. PWH were antiretroviral therapy (ART)-naive and were not on ART for more than 7 days in the preceding 6 months and had CD4^+^ T cell counts >200 cells/μL within 3 months of clinical visit. Exclusion criteria for both cohorts are extensively detailed elsewhere (33). In addition to blood samples, study participants underwent a flexible sigmoidoscopy with multiple colon pinch biopsies obtained. All study participants voluntarily gave written, informed consent. Cohort characteristics are noted in Table 1.

### RNAseq.

Total RNA was extracted from colon pinch biopsies using the Qiagen RNeasy minikit. An mRNA library was prepared from the extracted RNA using Lexogen QuantSeq 3’mRNA-Seq Library Prep Kit (FWD) for Illumina sequencing. Sequencing was performed at the Genomics & Microarray Core on the University of Colorado Anschutz Medical Campus using Illumina HiSEQ2500 for 150 × 2 pair-end read sequencing. An average of 41 million reads (range: 7.8–158 million) per study participant was obtained (37).

### RNASeq normalization, differential expression and pathway analysis.

After quality screening using FastQC, adaptors were removed using *cutadapt* and subsequently mapped to the human_grch38 genomic reference database using *Hisat*. FeatureCount (subread package) was used to extract raw gene expression counts for each sample. The genes which have at least five read counts per sample on average were kept for differential expression (DE) analysis. Reads were normalized using the TMM method (37, 38). The TMM method involves calculating a scaling normalization factor for each study participant by accounting for library size and observed counts, while under the assumption that majority of genes are not differentially expressed. The negative binomial model in edgeR (39) was used to identify significantly altered V genes between healthy controls and PWH. The FDR (false discovery rate) was controlled at 5% using the Benjamini and Hochberg method (40). All analyses were conducted with R (version 4.0.2) through RStudio (Version 1.2.1335). TMM-normalized data were also subjected to Ingenuity Pathway Analysis (IPA) (41) to determine pathways that were significantly altered in PWH versus controls. Individual genes of B cell-associated pathways were extracted from the IPA database and fold-differences were calculated by taking the ratio of the average TMM-normalized expression in PWH versus controls.

### Amplification of Ig genes for SHM analysis.

RNA (1 μg) was reversed transcribed using the Protoscript II First Strand cDNA Synthesis Kit (New England Biolabs). The sequencing library was prepared using a modified two-step overlap extension PCR method (Fig. 4A), in which the first PCR amplified either heavy or light chains from cDNA using multiplexed primers designed to capture 85–90% of heavy chain and light chain Ig expression (Table S1 in supplemental material). These primers also contained a barcode unique to each sample and a portion of the required Illumina sequence adaptors. The second PCR used an overlap extension strategy to add the remaining sequence necessary for Illumina sequencing. This strategy was adopted due to inefficient amplification when the full-length Illumina sequence adaptor was included in the primer used in the first PCR.

The first PCR used Phusion High-Fidelity DNA polymerase (New England Biolabs) with 10 μM multiplexed primers listed in Table S1 in supplemental material using the following conditions: 98°C for 1 min, followed by 35 cycles of (98°C for 30 s, 55°C for 15 s, 72°C for 17 s), with a 72°C final elongation step for 7 min. Amplicons were gel purified in a 1.5% agarose gel and extracted using a QIAquick Gel Extraction Kit (Qiagen), and 2 μL of purified amplicon was used in the second PCR using Phusion High-Fidelity DNA polymerase (New England Biolabs) as well as a forward overlap extension primer (10 μM) and a reverse overlap extension primer (10 μM):

5′-AATGATACGGCGACCACCGAGATCTACACTCTTTCCCTACACGACGCTC-3′

5′-CAAGCAGAAGACGGCATACGAGATGTGACTGGAGTTCAGACGTGTGC-3′

This second PCR utilized the following conditions: 98°C for 1 min, followed by 5 cycles of (98°C for 30 s, 55°C for 15 s, 72°C for 17 s), then 30 cycles of (98°C for 30 s, 61°C for 15 s, 72°C for 17 s) with a 72°C final elongation step for 7 min. Amplicons were gel-purified using the QIAquick Gel Extraction Kit (Qiagen). Samples were pooled together, normalizing the quantity of DNA from each sample using concentrations determined by a PicoGreen Assay (Thermo-Fisher), prior to loading in an Illumina MiSeq sequencer.

### SHM determination.

Sequences were compared to a custom database generated using IMGT (http://www.imgt.org) sequences (42). To capture Ab genes with high levels of SHM, we mapped V genes using a lower threshold of 70% sequence identity. Mutations were calculated by comparing differences between the generated sequence and the matching reference sequence.

### Microbiome analysis.

Laboratory and analytic methods used to profile the colon-associated microbiomes of study participants were described previously (33–36). In brief, bacterial profiles were generated from colon pinch biopsies by broad-range amplification and sequencing of bacterial 16S rRNA genes (V4 variable region). Illumina 2 × 250 paired-end reads were quality filtered, demultiplexed, merged, and classified using the SILVA Incremental Aligner (SINA 1.2.11) (43). A species name was assigned when a sequence overlapped the Silva database sequence by at least 95% sequence length with at least 99% sequence identity and the taxonomy of the database hit matched the taxonomy returned by SINA. Relative abundances (RA) of bacterial taxa were calculated as the number of sequences for a specific taxon standardized to the total number of sequences for the study participant. Species RA was calculated based on the fraction of all classified species detected within each individual. The effect of HIV-1 serostatus on the colon-associated microbial communities of the study participants have been extensively reported (33–36).

### Statistical analyses.

TMM normalized values were compared between PWH and controls. Multi-group comparisons were evaluated using one-way ANOVA with pairwise comparisons using the Tukey’s honest significant difference test, whereas two-group comparisons utilized either a 2-tailed parametric Student's *t* test or nonparametric Mann-Whitney U test depending on whether the sample distribution was normal (GraphPad Prism 8.0). Multivariable linear models were used to test the association between V gene expression and clinical parameters or microbiome relative abundance (RA). Linear models regressed V gene TMM values on microbiome RA, including an interaction term between microbiome RA and HIV-1 status, while adjusting for age, HIV-1 status, and sex. Similar models, without an interaction term or adjustment for HIV-1 status, were also used to test the association between the number of single mutations per unique sequence and clinical parameters or microbiome RA. The read counts were normalized then regularized log-transformed to serve as the outcome. The microbiome RA was at the phylum, family, genus, and species levels. Taxa with a minimum prevalence of 75% across participants and a minimum RA of 1% for at least one participant were included in regression analyses, and resulted in 4 phylum, 20 family, 24 genus, and 52 species. FDR was controlled at 5% by the Benjamini and Hochberg method. FDR corrections were implemented for each V gene at the family, genus and species levels separately. No multiple testing correction was made at the phylum level due to the small number of phyla included in regression analyses. Biostatistical analyses were conducted with R (version 4.0.2) through RStudio (Version 1.2.1335).

### Data availability.

Illumina sequences were deposited under NCBI BioProject PRJNA864820.
